# Does Encapsulation Protect Embryos from the Effects of Ocean Acidification? The Example of *Crepidula fornicata*

**DOI:** 10.1371/journal.pone.0093021

**Published:** 2014-03-26

**Authors:** Fanny Noisette, Thierry Comtet, Erwann Legrand, François Bordeyne, Dominique Davoult, Sophie Martin

**Affiliations:** 1 Sorbonne Universités, UPMC Univ. Paris 06, UMR 7144, Station Biologique de Roscoff, Roscoff, France; 2 CNRS, UMR 7144, Station Biologique de Roscoff, Roscoff, France; University of Western Sydney, Australia

## Abstract

Early life history stages of marine organisms are generally thought to be more sensitive to environmental stress than adults. Although most marine invertebrates are broadcast spawners, some species are brooders and/or protect their embryos in egg or capsules. Brooding and encapsulation strategies are typically assumed to confer greater safety and protection to embryos, although little is known about the physico-chemical conditions within egg capsules. In the context of ocean acidification, the protective role of encapsulation remains to be investigated. To address this issue, we conducted experiments on the gastropod *Crepidula fornicata.* This species broods its embryos within capsules located under the female and veliger larvae are released directly into the water column. *C. fornicata* adults were reared at the current level of CO_2_ partial pressure (*p*CO_2_) (390 μatm) and at elevated levels (750 and 1400 μatm) before and after fertilization and until larval release, such that larval development occurred entirely at a given *p*CO_2_. The *p*CO_2_ effects on shell morphology, the frequency of abnormalities and mineralization level were investigated on released larvae. Shell length decreased by 6% and shell surface area by 11% at elevated *p*CO_2_ (1400 μatm). The percentage of abnormalities was 1.5- to 4-fold higher at 750 μatm and 1400 μatm *p*CO_2_, respectively, than at 390 μatm. The intensity of birefringence, used as a proxy for the mineralization level of the larval shell, also decreased with increasing *p*CO_2_. These negative results are likely explained by increased intracapsular acidosis due to elevated *p*CO_2_ in extracapsular seawater. The encapsulation of *C. fornicata* embryos did not protect them against the deleterious effects of a predicted *p*CO_2_ increase. Nevertheless, *C. fornicata* larvae seemed less affected than other mollusk species. Further studies are needed to identify the critical points of the life cycle in this species in light of future ocean acidification.

## Introduction

Early life history stages of marine species, including embryos and larvae, are of crucial importance in population dynamics as they ensure dispersion, colonize new areas and sustain populations [1]. Their success in development and final recruitment are essential for the persistence of viable populations. Early stages of marine invertebrates are in general morphologically and ecologically distinct from the adult stage and are generally thought to be more sensitive to environmental stress [1] although, in some cases, they may be more tolerant than adults, e.g. some Antarctic species exposed to warming [2]. In the context of climate change, early development may be affected by various factors, such as temperature increases, hypoxia zones or ocean acidification. Due to the increase in atmospheric *p*CO_2_ predicted for the end of the century (from 475 to 1313 μatm according to the Intergovernemental Panel on Climate Change (IPCC)), pH in surface seawaters is likely to decline by 0.06 – 0.32 units [3], leading to a decrease in carbonate ion concentrations (CO_3_^2−^) and a reduction in the calcium carbonate saturation state (Ω) [4]. Due to these changes in seawater carbonate chemistry, ocean acidification is considered a major threat to calcifying marine species, affecting their physiology and impairing their ability to build calcium carbonate shells and skeletons [8], [9], [10], [11], which can ultimately modify their behavior and distribution [5], [6], [7]. Early life stages (embryos, larvae and juveniles) of calcifying species are thus expected to be highly affected by ocean acidification [12], [13], as opposed to non-calcified larvae which are predicted to be more tolerant [14], [15]. This relatively higher vulnerability is likely due to fragile larval skeletons [9] and their high ratio of exposed surface-to-body mass compared to adults [16]. Identifying life history stages that are the most vulnerable to global change is needed to determine bottlenecks for species persistence and addressing their sensitivity to acidification is a major issue in a changing ocean [9].

Responses to near-future (end of century) levels of *p*CO_2_ depend on species, populations, habitats and developmental stages [9], [17], [18], [19] and understanding these effects on the early life stages requires taking into account the complete developmental cycle, from egg to juvenile [12]. In particular, the impact of elevated *p*CO_2_/decreased pH on early life stages has been investigated in a broad range of species, including corals [20], [21], echinoderms [22], [23], [24], crustaceans [15], [16], [25], mollusks [26], [27], and fish [28], [29]. In mollusks, which have been studied intensively (see refs. [26], [30] for a review), deleterious effects of increased *p*CO_2_ have been demonstrated on fertilization success [31], [32], hatching success [33], [34], larval survival [35], [36], growth [37], [38], shell formation [39], [40], development duration [41], [42], and settlement [43], [44].

Most of the species studied are broadcast spawners, which may be considered particularly vulnerable to ocean acidification because fertilization and complete pelagic larval life occur in the water column [9], [45]. Whether alternative reproductive modes are affected in a changing ocean is still poorly documented. Brooding and/or egg laying in egg masses or capsules are typically assumed to confer protection to developing embryos [46], [47]. For example, it has been shown that encapsulated embryos of some gastropod species survive better in conditions of salinity stress than embryos removed from their capsule [48], [49]. A few studies have explored the effects of decreased pH on embryos brooded and/or laid in benthic gelatinous egg masses or in egg capsules in bivalve [50], gastropod [35], [41], [51], [52] or cephalopod [27], [53] mollusks. Depending on the study, reduced pH has different effects that are related to the range of species habitats and the strategy to protect embryos (brooding, egg masses, capsules) studied as well as the source of pH change (*p*CO_2_ increase, salinity stress). Encapsulation has been suggested to protect embryos against ocean acidification [41], [54], whereby the buffer capacity of intracapsular fluids may reduce the potential effect of extracapsular elevated *p*CO_2_ seawater.

To study this issue in a non-broadcast-spawner species, we chose the slipper limpet *Crepidula fornicata*, Linné 1758 (Gastropoda) as our biological model. Native to the northeast American coast, this species was introduced in Europe at the end of the 19^th^ century, primarily via oyster farming [55], and has now become invasive in bays and estuaries where it reaches very high densities of up to several thousands of individuals per m^2^ [56]. It has a bentho-pelagic life cycle, with a number of original features. Benthic adults form stacks with males at the top and females at the bottom. After internal fertilization, females brood their embryos in egg capsules for 3 to 4 weeks [57], [58]. Capsules are protected between the neck and the propodium of the female parent and attached to the substratum to which the female is fixed [58]. Each female spawns between 28 and 64 capsules, each containing 300 to 500 embryos [57]. At the end of capsular development, veliger larvae are released at a size of about 400 μm in length into the water column where they spend between 2 and 7 weeks [59], [60]. Upon reaching competence (800–1000 μm in length), larvae are able to metamorphose and settle on hard substrata [59], [60].

The objective of this work was to investigate the effects of near-future levels of *p*CO_2_ on the development of *C. fornicata* encapsulated embryos by studying the shell morphology and mineralization level of released larvae. To ensure that the complete development, from the egg to the released larva, occurred under high *p*CO_2_, parents were conditioned to the different *p*CO_2_ levels before mating occurred.

## Methods

### *Crepidula fornicata* adult collection and culture

*C. fornicata* stacks were collected by SCUBA divers on 30 November 2011, after the end of the reproductive period [61] in Morlaix Bay (northwestern Brittany, France), at the “Barre des Flots” site (3°53.015'W; 48°40.015'N). No specific permissions were required for sampling at the selected location, as it is not privately-owned or protected. Field sampling did not involve endangered or protected species.

After being held 6 weeks in natural ambient unfiltered seawater, *C. fornicata* adults were randomly distributed into 18 aquarium tanks of 10 L each (adapted from [62]) and reared for 24 weeks (12 January 2012 to 28 June 2012) in three *p*CO_2_ treatments selected according to the recommendations of Barry et al. [63]: (1) 390 μatm (pH on the total scale (pH_T_)  = 8.07) as the current *p*CO_2_ (control), (2) 750 μatm (pH_T_  =  7.82) and (3) 1400 μatm (pH_T_  =  7.56); the former two *p*CO_2_ levels are pessimistic scenarios predicted for the end of the century by the IPCC [3]. The *p*CO_2_ was adjusted by bubbling CO_2_-free air (current control *p*CO_2_) or pure CO_2_ (two elevated *p*CO_2_ treatments) in three 100 L header tanks supplied with unfiltered seawater pumped at the foot of the Station Biologique de Roscoff. Each of the three *p*CO_2_ treatments had six 10 L replicate aquaria. This was an open system, and CO_2_-treated seawater from the mixing header tanks was continuously supplied to the 18 aquaria (6 per *p*CO_2_ condition), at a rate of 9 L h^−1^ (i.e. a renewal rate of 90% h^−1^). Aquaria were placed in a thermostatic bath where temperature was controlled to within ±0.2°C using 150 to 250 W submersible heaters. *C. fornicata* adults were grown at four successive temperature levels (10, 13, 16 and 19°C) which corresponded to the range of *in situ* temperatures typically encountered in our study area (Service d’Observation de la Mer et du LITtoral data). Adults were reared for four weeks at each temperature level. Changes in temperature were implemented slowly, with increases of 0.2°C day^−1^ over a period of two weeks.

*p*CO_2_ and temperature were monitored and controlled by an off-line feedback system (IKS Aquastar, Karlsbad, Germany) that regulated the addition of gas in the header tanks and the on/off heater switch in the thermostatic bath. The pH values of the system were adjusted from daily measurements of pH_T_ in each of the 18 aquaria using a pH meter (826 pH mobile, Metrohm AG, Herisau, Switzerland) calibrated using Tris/HCl and 2-aminopyridine/HCl buffers [64]. Slipper limpets were fed three times a week with a mix made from a stock solution of *Chaetoceros gracilis* (∼15×10^6^ cells mL^−1^) and *Isochrysis affinis galbana* (∼26×10^6^ cells mL^−1^). This algal mix (400 mL) was distributed in each aquarium. Seawater flow was stopped for two hours to allow the limpets to feed.

Seawater parameters were monitored throughout the experiment in each of the 18 aquaria. pH_T_ and temperature were recorded daily. Total alkalinity was measured every four weeks, by 0.01 N HCl potentiometric titration on an automatic titrator (Titroline alpha, Schott SI Analytics, Mainz, Germany) in a 20 mL seawater sample taken from each aquarium. Salinity was also measured every four weeks with a conductimeter (LF 330/ SET, WTW, Weilheim, Germany) and it varied between 34.2±0.1 and 35.1±0.1 over the course of the experiment. The carbonate chemistry of seawater, i.e. dissolved inorganic carbon (DIC), exact *p*CO_2_ and saturation state of aragonite (Ω_Ar_) were calculated for each *p*CO_2_ and temperature treatment using CO_2_SYS software [65] with the constants of Mehrbach et al. [66] refitted by Dickson and Millero [67]. Mean values of these parameters are given in Table 1.

**Table 1:** Seawater parameters.

		Temperature		pH_T_		*p*CO_2_		A_T_		DIC		Ω_Ar_	
		(°C)				(μatm)		(μEq kg^−1^)		(μmol C kg^−1^)			
	n (except A_T_)	Mean	SE	Mean	SE	Mean	SE	Mean	SE	Mean	SE	Mean	SE
**10°C**													
390 μatm	23	9.7	0.2	8.14	0.01	323	7	2365	2	2138	4	2.47	0.04
750 μatm	23	9.8	0.2	7.82	0.01	729	19	2369	2	2270	4	1.33	0.03
1400 μatm	23	9.5	0.2	7.55	0.03	1487	75	2377	3	2366	11	0.78	0.08
**13°C**													
390 μatm	27	12.9	0.2	8.12	0.02	356	25	2418	2	2167	8	2.76	0.07
750 μatm	27	13.0	0.1	7.81	0.01	781	20	2416	2	2304	3	1.48	0.03
1400 μatm	27	12.8	0.1	7.53	0.01	1557	43	2422	2	2405	4	0.82	0.02
**16°C**													
390 μatm	28	15.9	0.1	8.08	0.01	376	11	2379	5	2127	5	2.80	0.05
750 μatm	28	16.1	0.1	7.82	0.00	748	8	2369	5	2238	2	1.66	0.01
1400 μatm	28	16.0	0.1	7.55	0.01	1492	19	2380	5	2345	2	0.94	0.01
**19°C**													
390 μatm	23	18.4	0.5	8.02	0.01	550	10	2391	2	2152	5	2.70	0.05
750 μatm	23	18.6	0.5	7.77	0.01	858	19	2395	3	2266	4	1.68	0.04
1400 μatm	23	18.4	0.5	7.51	0.01	1652	41	2394	3	2359	4	0.96	0.03

Legend: Mean parameters of carbonate chemistry in each *p*CO_2_ treatment at each temperature level. The pH on the total scale (pH_T_) was measured daily and total alkalinity (A_T_) was measured every 4 weeks. Other parameters were calculated with the CO_2_SYS software [65]. *p*CO_2_: CO_2_ partial pressure; DIC: dissolved inorganic carbon; Ω _Ar_: saturation state of aragonite.

### Larvae collection

In *C. fornicata*, at the end of the embryonic development, the capsule membrane splits and veliger larvae are released in seawater. To prevent released larvae from escaping from their source aquarium, 200 μm mesh size nets covered the overflow outlet of each aquarium. Offspring presence was checked visually every day to collect larvae within 24 h post-hatching. When present, larvae were collected by pouring aquarium seawater on a 200 μm mesh sieve, rinsed with seawater and preserved in 96% ethanol.

Pools of larvae from adults acclimated to the different *p*CO_2_ levels since January were collected from the different *p*CO_2_ conditions at the temperature level of 19°C between 8 and 24 June 2011. Only samples with enough intact larvae were used. Thus two viable samples per *p*CO_2_ condition were studied.

### Morphological variables

**Figure 1:**
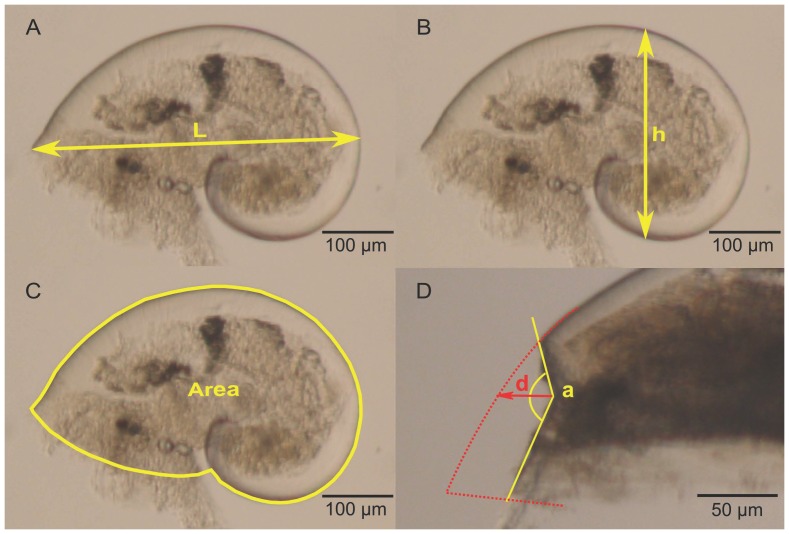
Morphological variables. All measurements were taken on larvae lying on their right side. A: maximum shell length (L) (in μm); B: maximum shell height (h) (in μm); C: projected surface area of the left side (in mm^2^); D: deformity index, a is the angle of the abnormality (in degrees) and d, the depth of the abnormality (in μm).

Morphological measurements were performed on a random subsample of 40 larvae when possible or at least 20 larvae from each of the 6 larval pools. Larvae with unbroken shells were isolated in sterile, flat-bottom, 96-well plates and preserved in pure glycerol as described in Auzoux-Bordenave et al. [68]. Each larva was placed on its right side and photographed under light microscopy using an Olympus Camedia C-7070 camera attached to an Olympus SZX 12 dissecting microscope. Pictures were taken without autofocus at ×90 magnification. Maximum length, height and projected surface area of the left side (Figures 1A, B, and C, respectively) were measured by analyzing images with ImageJ software [69], after calibration with a stage micrometer.

### Abnormalities

In each subsample used for morphological measurements, veliger larvae with abnormal shells were counted and the percentage of abnormal larvae was estimated per *p*CO_2_ treatment. To be considered as a shell abnormality and not as a broken shell, deformities had to be devoid of fracture lines. A “deformity index” was calculated to quantify the intensity of the shell deformity. It was defined as the ratio between the angle formed by the abnormality and its “depth”, which is the distance between the theoretical curve of the shell and the forest point (extreme point) of the deformity (Figure 1D).

### Shell mineralization

For each *p*CO_2_ treatment, 5 to 8 larvae were randomly chosen among the previous subsamples and observed under polarized light to determine birefringence patterns with an Olympus dissecting microscope equipped with polarizing filters. All polarized images were acquired with an Olympus camera at ×100 magnification with 40 ms light exposition. Birefringence under polarized light is due to the mineral phase composing the shell [40], [68], [70], [71]. In the absence of mineralized structures, there is no birefringence and the picture looks totally black. Under identical light conditions, areas appearing more birefringent contain a much larger proportion of crystalline calcium carbonate [70], [72]. The intensity of birefringence of each shell was used as a proxy for mineralization level for the three *p*CO_2_ treatments. It was quantified from pictures by using ImageJ software [69]. Pictures of polarized shells were first transformed into grayscale images. A mean gray value (in pixels) was determined for each birefringent zone. All birefringent zones of the shell were compiled to obtain a global mean gray value, giving the intensity of the birefringence of the whole shell.

### Statistics

All statistical analyses were performed using the free software R 2.15.0 version [73]. Normality and homoscedasticity of the data were first checked using Shapiro and Levene tests, respectively. Due to the non-normality and heterogeneity of variance, the influence of *p*CO_2_ on morphological variables, deformity indices and birefringence intensity was analyzed using the non-parametric Kruskal-Wallis test followed by the Dunn post-hoc test [74]. A Chi-squared (χ^2^) test followed by G-tests (likelihood-ratio test) [75] were used to compare percentages of anomaly between the three *p*CO_2_ conditions.

## Results

### Morphological variables

**Figure 2:**
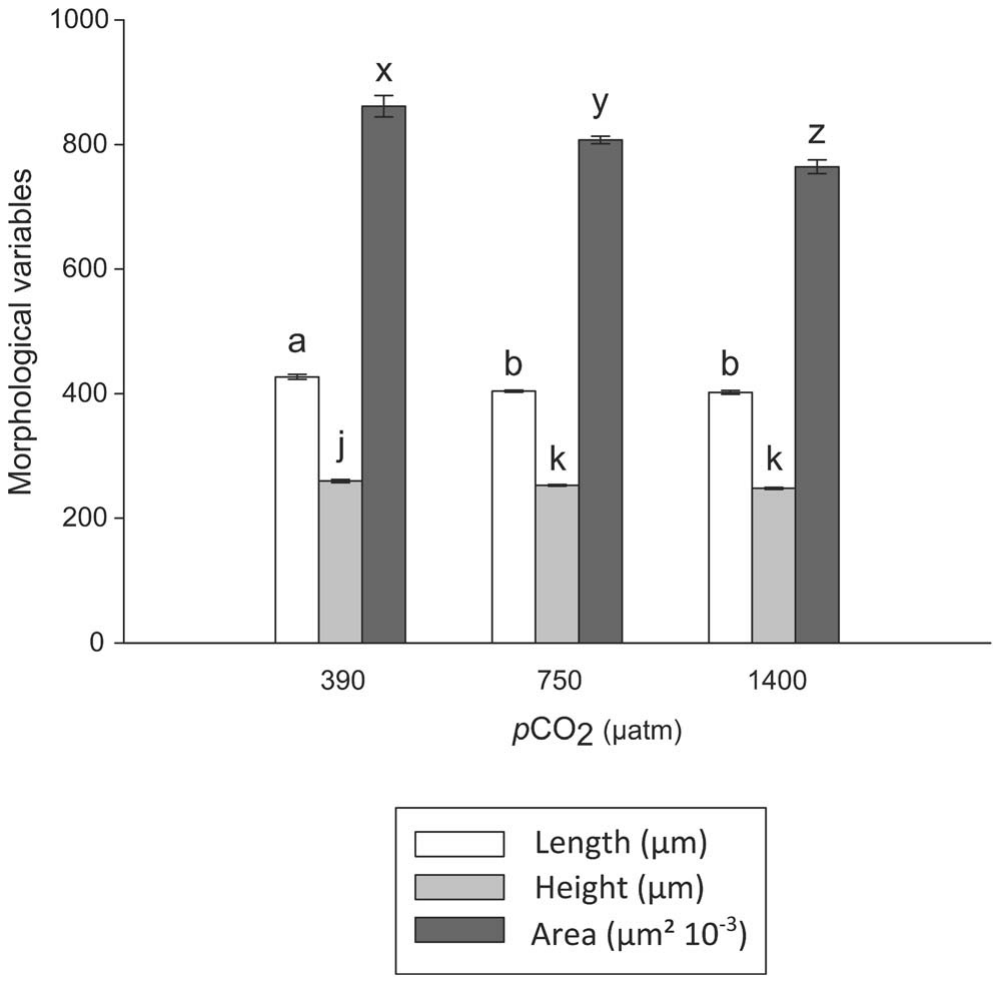
Morphological variables. Mean shell length, height and left surface area (± SE) in the different *p*CO_2_ treatments. Different letters above bars indicate significant differences between treatments (p<0.05, Dunn post-hoc test), n = 51 to 92.

*p*CO_2_ significantly affected length, height and surface area of the hatched larvae (Figure 2, Table 2). These morphological variables are related to each other and were generally influenced in the same way by *p*CO_2_. Length and height were the highest at 390 μatm and significantly decreased with increased *p*CO_2_. Larvae collected at 750 and 1400 μatm *p*CO_2_ showed a decrease of 5.3% and 5.9% in length, respectively, and 2.6% and 4.5% in height, respectively, compared to control larvae (390 μatm). Similarly, the greatest shell surface area was observed at 390 μatm *p*CO_2_, but then significantly decreased by 6.2% and 11.2% at 750 and 1400 μatm *p*CO_2_, respectively.

**Table 2:** Effect of *p*CO_2_ on morphological variables, abnormality indices and intensity of birefringence.

	Kruskal-Wallis Test		
	df	H	p
Length	2	37.353	< 0.001
Height	2	16.235	< 0.001
Surface area	2	30.106	< 0.001
Abnormality index	2	6.046	0.049
Intensity of birefringence	2	14.562	< 0.001

Legend: Summary of the non-parametric Kruskal-Wallis tests testing the effect of *p*CO_2_ on each morphological variable, abnormality index and birefringence intensity.

### Abnormalities

**Figure 3:**
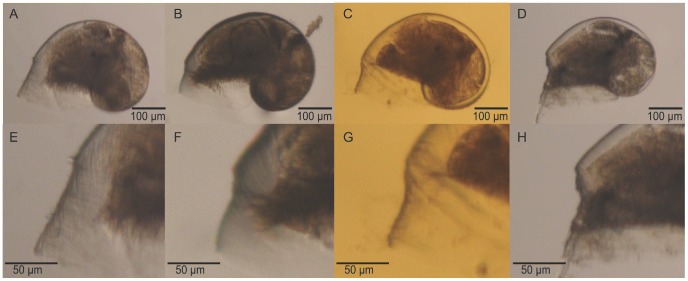
Shell abnormalities. Different intensities of shell abnormalities observed among samples. A, B, C, and D show whole larvae whereas E, F, G, and H show the detail of their respective abnormalities.

**Figure 4:**
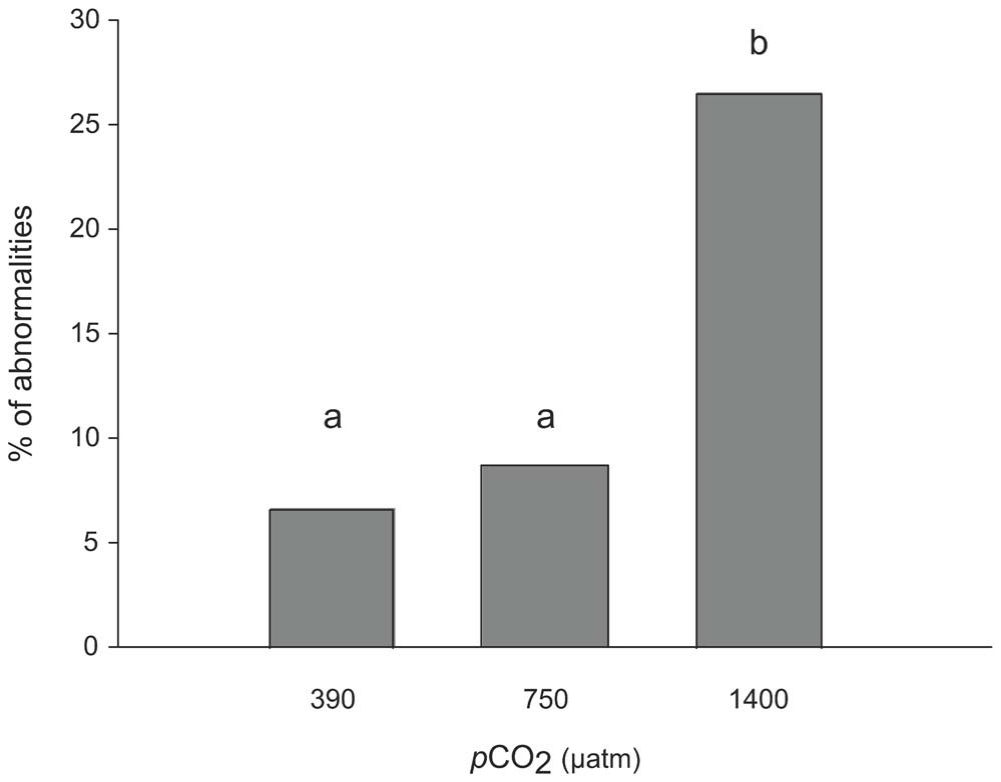
Percentage of abnormalities in the different *p*CO_2_ conditions. Different letters above bars indicate significant differences between treatments (G-test, p<0.05).

Abnormalities in larvae were observed as notches located close to the shell aperture (Figure 3). The percentage of abnormal larvae increased with increased *p*CO_2_ and ranged from 6.7 to 26.5% (Figure 4; χ^2^ test, p<0.05). Abnormalities were 1.5- and 4-fold at *p*CO_2_ levels of 750 and 1400 μatm, respectively, than at 390 μatm. Furthermore, different intensities of shell abnormalities were observed with variation in notch acuteness. The deformity index varied between 0.03 (390 μatm *p*CO_2_) and 0.17 (1400 μatm *p*CO_2_). Although the Kruskal-Wallis test showed a marginally significant *p*CO_2_ effect (p = 0.049; Table 2), the pairwise Dunn post-hoc test did not detect significant differences between the three *p*CO_2_ treatments (p > 0.05).

**Figure 5:**
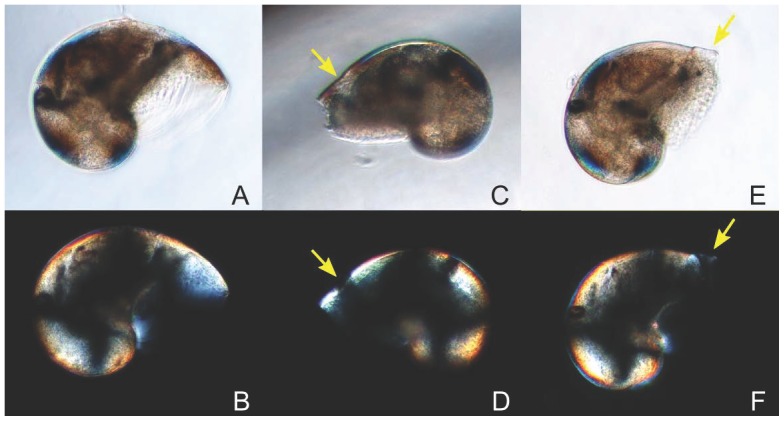
Shell abnormalities under normal (A, C, and E) and polarized (B, D, and F) light. A-B are pictures of a normal larva presenting the characteristic black cross of birefringence. C-D and E-F show examples of abnormalities observed among the samples, with arrows indicating less calcified zones.

Among the abnormal larvae observed under polarized microscopy (see below), some showed abnormalities which appeared less birefringent, and even not mineralized, as revealed by the lack of birefringence in these parts of the shell (Figure 5).

### Shell mineralization

**Figure 6:**
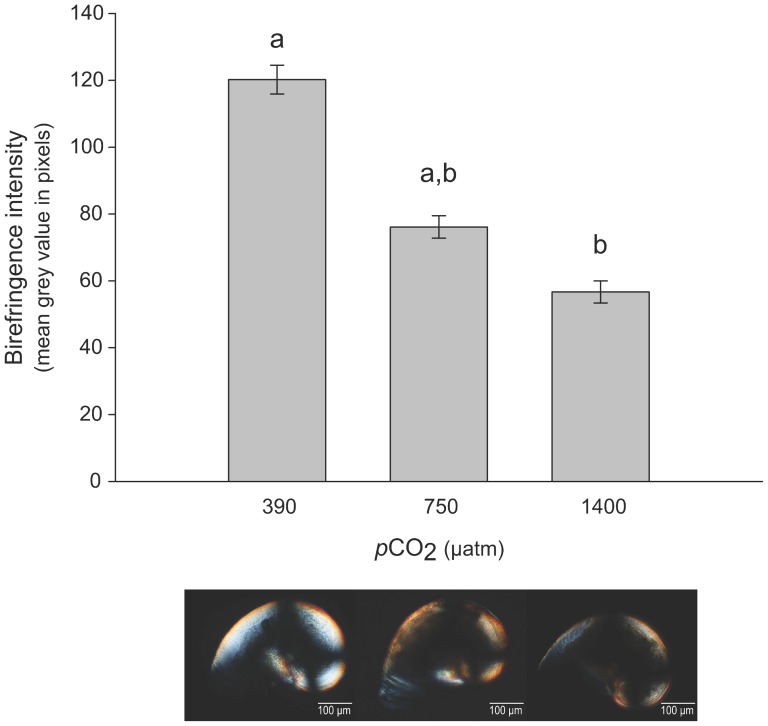
Birefringence intensity. Mean gray value (in pixel) of the shells under polarized light in the different *p*CO_2_ treatments (± SE). Bars with different letters are significantly different (Dunn post-hoc test, p<0.05,), n = 5 to 8 larvae per condition. Pictures taken under polarized light (below the graph) show birefringent patterns in the different *p*CO_2_ treatments.

Pictures taken under polarized light (Figure 6) suggest that the intensity of the birefringence decreased with increased in *p*CO_2_. The measure of birefringence intensity using the mean gray values estimated for each shell clearly confirmed this relationship (Table 2). Mineralization was greatest at 390 μatm *p*CO_2_, intermediate at 750 μatm *p*CO_2_ and lowest at 1400 μatm *p*CO_2_ (Figure 6).

## Discussion

In our study, the effects of elevated *p*CO_2_ were integrated from embryo formation and throughout embryonic stages up until the release of veliger larvae. The integration of *p*CO_2_ effects across all developmental stages, from fertilization to settlement and beyond, is particularly instructive because early life stages may respond to environmental stressors in a different way than adults. Acute exposures of early life stages have shown various effects in growth or feeding performances [76], [77], but these may not represent field conditions. Results obtained from acclimation to high *p*CO_2_ across different life stages often differ from those arising from acute exposure of a given stage. For example, it has been shown that oyster D-veliger larvae grown from eggs fertilized at elevated *p*CO_2_ were more drastically affected than those first produced at ambient *p*CO_2_ and then reared later (embryo stages) at high *p*CO_2_ [78]. Keeping the parents under different *p*CO_2_ conditions before fertilization and until larval release allowed embryos develop entirely under a given level of stress. To our knowledge, only Dupont et al. [79] on sea urchins, Parker et al. [80] on mollusks and Vehmaa et al. [81] on copepods acclimated adults to high *p*CO_2_ during reproductive conditioning before studying larvae in the same *p*CO_2_ conditions.

*p*CO_2_ effects were first investigated on the shell morphology of the hatched larvae. The size (length, height and surface area) of the released larvae decreased with increased *p*CO_2_. Length and height were not significantly different between the *p*CO_2_ levels of 750 and 1400 μatm, whereas the shell surface area progressively decreased with increased *p*CO_2_ from 390 to 1400 μatm. Although in some rare cases, elevated *p*CO_2_ does not affect larval morphology and growth [82], [83], a correlation between high *p*CO_2_ and smaller size has been demonstrated in most bivalve and gastropod species studied to date (see review in Table 3), with *p*CO_2_ sometimes affecting the shape of the larval shell [41]. As observed here for length and height (ca. –5% in the two high *p*CO_2_ treatments), shell lengths of *Crassostrea gigas* veliger larvae are smaller under elevated *p*CO_2_, but are similar in conditions of pH lowered by 0.4 and 0.7 pH units, a range corresponding to our pH values [32]. Smaller size and delayed shell growth can be attributed to problems in shell deposition, delayed periostracum formation and/or increased shell dissolution, as hypothesized by Watson et al. [84].

**Table 3:** Literature review of reports of high *p*CO_2_ effects on morphological variables in mollusk larvae.

Reproductive mode	Species (veliger stage)	Measured parameter	pH conditions	*p*CO_2_ conditions (μatm)	% decrease due to *p*CO_2_	Study
Broadcast spawner	*Crassostrea gigas*	shell length	pH_NBS_ 7.4	2268	decrease	[40]
Broadcast spawner	*Crassostrea gigas*	shell length	pH_NBS_7.8	1000	16%	[78]
Broadcast spawner	*Crassostrea gigas*	shell length and height	pH_NBS_ 7.76 – 7.37	1386 – 3573	10.6%	[32]
Broadcast spawner	*Crassostrea gigas*	shell area	pH_NBS_ 7.7 – 7.4	1497 – 2386	18.7 – 29%	[101]
Broadcast spawner	*Crassostrea virginica*	shell length	pH_T_ 7.84 – 7.49	≈ 650 – 1500	16.7%	[38]
Broadcast spawner	*Crassostrea virginica*	shell area	pH_NBS_ 7.76	840	16%	[94]
Broadcast spawner	*Saccostrea glomerata*	shell length	-	1000	22%	[31]
Broadcast spawner	*Saccostrea glomerata*	shell length	pH_NBS_7.8	1000	34%	[78]
Broadcast spawner	*Saccostrea glomerata*	shell length	pH_NBS_7.9	856	31.6 – 1.3%	[80]
Broadcast spawner	*Saccostrea glomerata*	shell length	pH_NBS_ 7.8 – 7.6	508.8 – 775.6	8.7 – 6.3%	[84]
Broadcast spawner	*Saccostrea glomerata*	shell height	pH_NBS_ 7.8 – 7.6	508.8 – 775.6	7.5 – 5.1%	[84]
Broadcast spawner	*Mytilus californianus*	shell area	pH_NBS_ 7.75	970	5 – 7%	[37]
Broadcast spawner	*Mytilus edulis*	shell length	pH_NBS_ 7.8	1200	4.5 – 6%	[39]
Broadcast spawner	*Mytilus edulis*	shell thickness	pH_NBS_ 7.8	1200	12%	[39]
Broadcast spawner	*Mytilus edulis*	shell area	pH_NBS_ 7.6	1388 – 1493	7 – 8%	[16]
Broadcast spawner	*Mytilus galloprovincialis*	shell length and height	pH_NBS_ 7.4	2000	26 – 20%	[86]
Broadcast spawner	*Argopecten irradians*	shell length	pH_T_ 7.84 – 7.49	≈ 650 – 1500	50%	[38]
Broadcast spawner	*Argopecten irradians*	shell diameter	pH_T_ (?) 7.5	1500	30.7%	[43]
Broadcast spawner	*Argopecten irradians*	shell thickness	pH_T_ (?) 7.5	1500	43%	[43]
Broadcast spawner	*Argopecten irradians*	shell length	pH_SW_ 7.39	1987	18.9 – 7.5%	[102]
Broadcast spawner	*Pecten maximus*	shell length	pH_NBS_(?) 7.51	1627	10%	[85]
Broadcast spawner	*Pecten maximus*	shell height	pH_NBS_(?) 7.51	1627	5%	[85]
Broadcast spawner	*Macoma balthica*	shell length	pH_NBS_ 7.7 – 7.2	1700 – 4400	4.3 – 8.5%	[103]
Broadcast spawner	*Macoma balthica*	shell length	pH_NBS_ 7.8 – 7.5	-	16.2 – 16.9%	[42]
Broadcast spawner	*Mercenaria mercenaria*	shell length	pH_T_ 7.84 – 7.49	≈ 650 – 1500	22.7%	[38]
Broadcast spawner	*Mercenaria mercenaria*	shell diameter	pH_T_ (?) 7.5	1500	25.5%	[43]
Broadcast spawner	*Mercenaria mercenaria*	shell thickness	pH_T_ (?) 7.5	1500	43.0%	[43]
Broadcast spawner	*Haliotis discus hannai*	shell length	pH_T_ (?) 7.71	1050	2.50%	[33]
Broadcast spawner	*Haliotis discus hannai*	shell length	pH_NBS_ 7.6 – 7.3	1224.6 – 2543.6	decrease	[34]
Broadcast spawner	*Haliotis kamtschatkana*	shell length	pH_NBS_(?) 8.07	800	5%	[36]
Brooding in pallial cavity	*Ostrea chilensis*	shell thickness	pH_NBS_(?) 7.7 – 7.0	-	no change	[50]
Brooding in pallial cavity	*Ostrea lurida*	shell growth	pH_T_ 7.76	1000	5 – 14%	[104]
Egg masses	*Littorina obtusata*	shell length	pH_NBS_(?) 7.6	-	10%	[41]
Egg masses	*Stylocheilus striatus*	shell area	pH_NBS_ (?) 7.6	-	24 – 36%	[52]
Egg	*Sepia officinalis*	total weight	pHT 7.84 – 7.60	750 – 1430	no change	[53]
Encapsulation + brooding	*Crepipatella dilatata*	shell thickness	pH_NBS_ (?) 6	-	30%	[51]
Encapsulation + brooding	*Crepidula fornicata*	shell length	pH_T_ 7.8 – 7.6	750 – 1400	5.3 – 10.7%	Present study
Encapsulation + brooding	*Crepidula fornicata*	shell height	pH_T_ 7.8 – 7.6	750 – 1400	2.6 – 13.1%	Present study
Encapsulation + brooding	*Crepidula fornicata*	shell area	pH_T_ 7.8 – 7.6	750 – 1400	6.2 – 19.8%	Present study

pH_NBS_: pH on the NBS scale; pH_T_: pH on the total scale; pH_SW_: pH on the seawater scale.

Such processes may lead to developmental abnormalities and to an increase in their frequency under elevated *p*CO_2_. Some (7%) *C. fornicata* larvae in the control group (390 μatm *p*CO_2_) showed mild shell abnormalities in the form of a notch close to the aperture. The frequency of this abnormality increased under high *p*CO_2_, being 1.5-fold more frequent at 750 μatm *p*CO_2_ and reaching 26% at 1400 μatm *p*CO_2_. The intensity of shell abnormality, estimated using the deformity index, did not vary significantly with increasing *p*CO_2_, although more pronounced shell deformities were detected at the highest *p*CO_2_ condition (1400 μatm). The occurrence of abnormal shells is a common response in mollusk larvae exposed to elevated *p*CO_2_. In bivalves for example, abnormalities can occur as shell hinge and edge deformities [85], irregular-shaped shells [40] or protruding mantles [86]. The frequency of abnormalities can reach 40% of shell deformities in *Pecten maximus* larvae reared at 1250 μatm *p*CO_2_ [85], and even 90% of abnormal D-veligers in *Saccostrea glomerata* at 1000 μatm *p*CO_2_ [31]. In gastropods, larval shells are considered abnormal when shells are too small to fully cover the soft body [34] or when dissolution zones are observed at the edge of the aragonitic larval shell [33], with frequencies of abnormality ranging from 20% in *Haliotis discus hannai* at 1650 μatm *p*CO_2_ [33] to 40% in *Haliotis kamtschatkana* at 800 μatm *p*CO_2_ [36]. At extremely high *p*CO_2_ (> 1700 μatm), some abalone larvae are even unable to precipitate a calcareous shell [36], [44].

Such abnormalities may be due to different processes: (i) the production of amorphous CaCO_3_ may be affected by damage to embryonic ectodermic cells and/or (ii) seawater corrosion may induce shell dissolution, affecting the strength and calcification of some parts of the shell [32]. Here, the mineralization level of larval shells was investigated at each *p*CO_2_ level by observing the veliger aragonitic shell under polarized light [70]. The characteristic dark cross observed in each larval shell indicated a radial arrangement of aragonite crystals [72] and did not have been considered as non-crystalline zones. The intensity of birefringence was used as a proxy for mineralization because increases in birefringence reflect increases in crystalline structure and calcification of the shell. Observed under polarized light, abnormalities appeared less birefringent than the rest of the shell, suggesting that deformities were likely less calcified as proposed by Barros et al. [32]

The birefringence intensity of the larval shells decreased with increased *p*CO_2_, and was significantly lower at 1400 μatm *p*CO_2_. This drop in birefringence revealed a decrease in calcification, which may be related to a less mineralized matrix [87], or more likely to a reduction in shell thickness [72]. Our data did not allow us to discriminate between these two possibilities, but previous studies have already reported a decrease in shell thickness under high *p*CO_2_ in bivalve larvae. For example, using scanning electron microscopy measurements, Gazeau et al. [39] showed a decrease in thickness of 12% in *Mytilus edulis* larvae at 745 μatm *p*CO_2_. Talmage and Gobler [43] report a decrease in thickness of *Mercenaria mercenaria* (–43%) and *Argopecten irradians* (– 47.5%) larval shells after 17 days of development at 1500 μatm *p*CO_2_, which was associated with an impact on the integrity and the connectedness of the hinge structure. A decrease of 5.7% in shell thickness of brooded larvae of the oyster *Ostrea chilensis* has also been observed following a decrease in pH (down to 6.56) within the mother’s pallial cavity due to valve closure under salinity stress [50].

The decrease in larval size and mineralization level of the shell may be due to reduced CaCO_3_ saturation or hypercapnic suppression of metabolic pathways involved in the calcification process [9]. Very little is known about the conditions occurring during intracapsular development and how acidified seawater can affect encapsulated embryos. Previous studies have shown that egg capsules of some gastropods, including *C. fornicata*, are permeable to water and ions (e.g. [48], [71], [88]) and it can be assumed that the capsule wall in *C. fornicata* is almost impermeable to gas because of its low O_2_ conductance [89]. Under “normal” conditions, embryos of *C. fornicata* will progressively be exposed to hypoxia [89] and hypercapnia via their respiration. This may lead to low intracapsular pH, as reported in other gastropod species [90], without altering development [57]. Under elevated *p*CO_2_, diffusion of more protons (H^+^) from external seawater to the intracapsular medium may alter intracapsular carbonate chemistry, thus enhancing metabolic acidosis. Similar acidosis can be observed under low salinity stress. For example, a decrease in pH to 6.4 recorded within the pallial cavity of the calyptraeid *Crepipatella dilatata* [47],[51] led to the partial shell decalcification of the brooded encapsulated embryos [51]. This decalcification may cause the release of some carbonate ions (CO_3_^2−^), which could bind to free H^+^ to form bicarbonate ions (HCO_3_^−^), thus buffering the intracapsular acidosis and limiting the drastic pH effects on larval metabolism. This potential buffering role, in combination with a decrease in the CaCO_3_ saturation state, is likely to affect shell mineralization and calcification. Such processes have been suggested to buffer acidosis resulting from anaerobiosis in *C. fornicata* [91], and may also explain our observations. Alternative mechanisms that can decrease the intracapsular acidosis, such as the active excretion of H^+^ out of the capsule through a proton pump, as shown in the cephalopod *Sepia officinalis* [92], need to be investigated.

Altogether, our results show that, despite the potential protective role provided by encapsulation and brooding, elevated seawater *p*CO_2_ affected the shells of the released larvae in *C. fornicata.* Embryos of *C. fornicata* were affected by high *p*CO_2_ during their intracapsular development. However, the overall low abnormality rate and low decrease in size suggested they were likely less affected than other mollusk early life stages. The natural exposure of embryos to low intracapsular pH as demonstrated in cephalopod eggs (pH on the seawater scale of the perivitalline fluid of ca. 7.35 at 16°C [53]) and gastropod capsules (pH of the intracapsular fluid lower than 7 [90]) could confer to *C. fornicata* larvae some resilience to elevated *p*CO_2_ levels. Indeed, it has been shown that bivalves naturally exposed to high *p*CO_2_ conditions in their habitat (due to high levels of benthic respiration or to seawater naturally enriched in CO_2_) are less affected by ocean acidification than other mollusk species [93], [94]. Further studies are however needed to determine the pH of the intracapsular fluid in *C. fornicata*, and how it will be affected under future scenarios of ocean acidification.

The effects of elevated *p*CO_2_ observed on *C. fornicata* larvae released from capsules suggest critical ecological consequences for their subsequent planktonic life and benthic settlement. Production of smaller larvae with weaker shell strength may increase vulnerability of larvae to predation and physical damages [37]. Furthermore, larvae physiologically stressed during their development by various abiotic factors may delay metamorphosis and settlement [94], staying longer in the water column which lead them to be more exposed to predators and diseases [94], [95], [96]. In addition, reduced size in early developmental stages may affect the juvenile survivorship and fitness [97], [98]. Given these consequences on the early life stages of *C. fornicata*, *p*CO_2_ may influence its invasion dynamics in its introduction range via reproductive success, larval survival and dispersal, and settlement success. Further studies are required to fully understand the interactions between climate change and biological invasions [99], [100]. In particular, more studies on early life stages and particularly the transition processes between them (e.g. metamorphosis) are needed to identify the potential tipping points, the demographic bottlenecks and the global resistance of non-native species in the context of ocean acidification.
